# The phylogenetic significance of leaf anatomical traits of southern African *Oxalis*

**DOI:** 10.1186/s12862-016-0792-z

**Published:** 2016-10-22

**Authors:** Michelle Jooste, Léanne L. Dreyer, Kenneth C. Oberlander

**Affiliations:** 1 Department of Botany and Zoology, University of Stellenbosch, Private Bag X1, Matieland, 7602 South Africa; 2 Department Conservation Ecology and Entomology, Stellenbosch University, Private Bag X1, Matieland, 7602 South Africa; 3 Institute of Botany, Academy of Sciences, Průhonice, CZ 252 43 Czech Republic

**Keywords:** Ancestral state reconstruction, Epistomaty, Leaf anatomy, *Oxalis*, Phylogenetics, Stomatal position

## Abstract

**Background:**

The southern African *Oxalis* radiation is extremely morphologically variable. Despite recent progress in the phylogenetics of the genus, there are few morphological synapomorphies supporting DNA-based clades. Leaflet anatomy can provide an understudied and potentially valuable source of information on the evolutionary history and systematics of this lineage. Fifty-nine leaflet anatomical traits of 109 southern African *Oxalis* species were assessed in search of phylogenetically significant characters that delineate clades.

**Results:**

A combination of 6 leaflet anatomical traits (stomatal position, adaxial epidermal cells, abaxial epidermal cells, mesophyll, sheath around vascular tissue, degree of leaflet conduplication) clearly support various clades defined by previous DNA-based phylogenetic work. Other, mostly continuous leaflet anatomical traits were highly variable and showed less phylogenetic pattern.

**Conclusions:**

Major and unexpected findings include the transition from ancestral hypostomatic leaflets to adaxially-located stomata in the vast majority of southern African *Oxalis*, the loss of semi-swollen AB epidermal cells and the gain of swollen adaxial and abaxial epidermal cells in selected clades, and multiple changes from ancestral bifacial mesophyll to isobilateral or homogenous mesophyll types. The information gathered in this study will aid in the taxonomic revision of this speciose member of the Greater Cape Floristic Region and provide a basis for future hypotheses regarding its radiation.

**Electronic supplementary material:**

The online version of this article (doi:10.1186/s12862-016-0792-z) contains supplementary material, which is available to authorized users.

## Background

Angiosperms show a great wealth of external morphological traits used as a classical data source to delineate groups in plant taxonomy [1–5], although in some plant groups (e.g. Scrophulariaceae [6, 7], morphological variation and homoplasy have made the designation of clades very difficult. Although much of our current understanding of angiosperm relationships has been achieved through analysis of DNA data, there is still great potential to find phylogenetically informative morphological characters in many angiosperm groups, particularly in groups that have been morphologically poorly-studied. In particular, plant anatomy has been considered of demonstrable and under-utilised benefit in systematic studies of plants, with the leaf proposed as “perhaps anatomically the most varied organ of angiosperms” [1].

*Oxalis* L. is by far the largest genus in the Oxalidaceae, including more than 500 species [8, 9]. This morphologically variable genus includes shrubs, stem succulents, herbs, annuals and geophytes, with a cosmopolitan distribution [5, 10–12]. Two centres of diversity for *Oxalis* are known, one in South America and the other in southern Africa [13, 14]. Approximately 220 *Oxalis* species and about 270 taxa [8, 15] have been recorded from southern Africa, with more than 90 % of these species endemic to South Africa [16]. Within southern Africa, *Oxalis* is by far the most diverse and has the highest number of endemic species within the Greater Cape Floristic Region (GCFR) [17]. All members of the southern African *Oxalis* lineage are geophytic [18], in contrast to approximately 31 % of the species from the South American diversity centre [9].

The southern African *Oxalis* radiation is extremely morphologically variable and its size and complexity have contributed to this lineage being poorly studied. Few traits are known as potential synapomorphies supporting clades [19] and systematic relationships among clades are poorly understood. The last major taxonomic work on South African *Oxalis* in a global context within the Oxalidaceae was by Knuth (1930) [20]. Salter (1944) [8] published the most comprehensive taxonomic revision of the southern African *Oxalis*. Unfortunately he only studied characters visible to the naked eye or through light microscopy (e.g. leaf-, stem- and bulb morphology) which was a limiting factor acknowledged by the author himself [8]. Dreyer (1996) [21] presented a detailed review of the palynology of southern African members of *Oxalis* and identified four major pollen types based on tectum structure. Despite the well-defined and easily-recognisable pollen type groupings [21], there was poor congruence between the morphological classification proposed by Salter (1944) [8] and the palynological classification of Dreyer (1996) [21]. The most recent major study on southern African *Oxalis* species, a large scale DNA sequence-based phylogeny, was published by Oberlander et al. (2011) [19]. Three molecular markers and three different inference methods were used to examine the phylogenetic relationships in a study that included three-quarters of all indigenous southern African *Oxalis* species and 14 outgroup species [19]. All southern African *Oxalis* taxa from formed a clade, and despite substantial incongruence between different molecular markers, the study resolved some previously unclear basal relationships between the major southern African lineages. The phylogeny proposed by Oberlander (2009) [22] was incongruent with the morphology-based taxonomy of Salter (1944) [8], but corresponded well with previous phylogenetic studies [23], and with the palynological classification by Dreyer (1996) [21]. Incongruence between molecular phylogenies and morphology-based taxonomies in *Oxalis* as a whole [19, 24, 25] has highlighted the need for more data from other disciplines such as anatomy, karyology, reproductive biology and ecology. Progress has been made on several of these fronts, including studies on the reproductive biology of *O. alpina* Savign. [26–28], work on population genetics and reproductive biology of rare and endangered *Oxalis* [29], tristyly [30], pollination biology [31], flowering phenology [32], pollen and ovule development [33] and chromosomal surveys in South American *Oxalis* [34, 35]. However, research into the anatomical diversity displayed by *Oxalis* has remained lacking.

Very few papers have focussed on *Oxalis* leaf anatomy and this has seldom involved more than a handful of species. Despite this limited research, results have often showed surprising variability, consistent with the tremendous variation in morphological attributes such as number and shape of leaflets, leaf and leaflet size, degree of leaflet conduplication, petiole length and shape, nature of the epidermis and indumentum attributes [8]. *Oxalis* leaves can be divided into three main regions: a semi-amplexicaul basal region, a petiole and a lamina, which is divided into one to multiple leaflets [8, 35]. Articulations separate the three main regions of the leaf, which allow nastic movement of the petiole and leaflets [8, 36–38]. Leaflet number has been considered a taxonomically useful morphological trait in *Oxalis* - the majority of *Oxalis* species have three leaflets per leaf [8, 9, 13]. Three to 13 leaflets per leaf have been recorded in the New World sect. *Ionoxalis* [13] and sect. *Palmatifoliae* [39] and up to 29 leaflets per leaf in southern African *Oxalis* [8]. Various comparative leaf anatomical studies include epidermal pavement cell types [40–42]: sinuous [43], angular [44] and papillose epidermal cell types have been reported in South American and European *Oxalis* species [45, 46]. Bladder epidermal cells have been observed on the abaxial (AB) leaflet surface of South American *O. carnosa* Molina [47] and swollen epidermal cells have been described from southern African *Oxalis* [8]. Taxonomic work on *Oxalis* section *Ionoxalis* reported that pubescence, trichome-types, -densities and -lengths were taxonomically significant traits, but considerable variation of trichome traits on the plant in general, and leaves specifically, for species in section *Ionoxalis* [13] and section *Corniculatae* [48] have been recorded. Hypostomatic leaflets have been documented among North American [13] and South American *Oxalis* taxa [47, 49]. A single known record of amphistomatic leaflets in *Oxalis* is known in *O. latifolia* [49], but stomatal distribution appears different between adaxial (AD) and AB surfaces in this study – AD stomata appear to be sparse and confined to above the midrib vascular tissue ([49], Figs. 2 and 3). Anomocytic and paracytic stomatal complex types have been reported in the Oxalidaceae [3, 49, 50]. To our knowledge, mesophyll arrangement types have only been recorded in three South American *Oxalis* species [49, 51], which showed the typical angiospermous bifacial condition and bifacial and isobilateral mesophyll arrangement types have been recorded in four southern African *Oxalis* species [52]. *Oxalis* is well-known for its high concentrations of calcium oxalate present in leaf tissue [53–56]. Trends of calcium oxalate deposits in some *Ionoxalis* species have been described, but these traits were not as taxonomically useful as previously assumed [13]. Pinnate venation types within leaflets were recorded in Oxalidaceae in general [53] and in sect. *Ionoxalis* [13]. The trait of a protruding vascular mid-rib has been recorded in three South American species [49] and the presence of sheaths have been described around the periphery of the vascular bundles in *O. corniculata* [51].

From the above-mentioned leaflet anatomical literature, it is obvious that there is scope for much more in-depth study of *Oxalis* leaflets in general and southern African *Oxalis* in particular, and there are doubtless many traits that have not yet been assessed or reported in the available literature. It is reasonable to expect that *Oxalis* may display systematically significant leaflet anatomical traits that may further clarify relationships between the southern African taxa. Our main objective was to systematically investigate leaflet anatomical traits across a broad phylogenetic spectrum of southern African *Oxalis*, and to determine which of these traits can be used to support the monophyly of southern African *Oxalis* clades [19].

## Methods

**Fig. 1:**
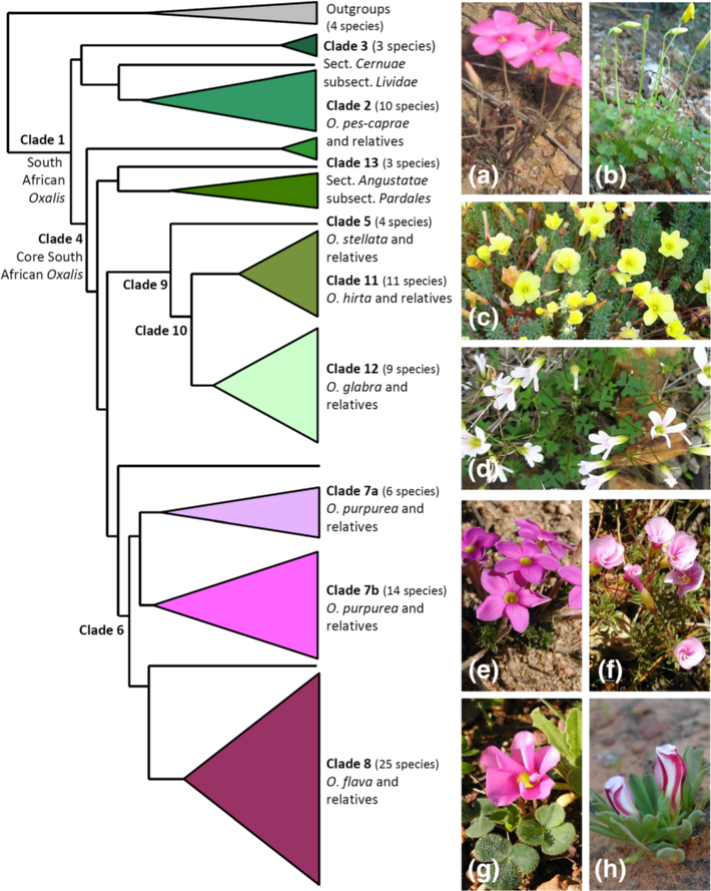
Summary of strongly supported *Oxalis* clades represented on the ITS phylogenetic tree from our study. Numbered clades correspond to those described by Oberlander et al. (2011) [19] and as mentioned in the text and figures throughout this article. Basal relationships amongst the Clade 4 lineages are unresolved in this ITS phylogenetic tree. The annotations of number of species refer to the number included in our study, which is approximately 50 % of all southern African *Oxalis*. Photographs of southern African *Oxalis* plants (**a**) *O. livida* from Clade 3, **b** *O. haedulipes* from Clade 2, **c** *O. grammophyla* from Clade 13, **d** *O. stellata* from Clade 5, **e** *O. hirta* from Clade 11, **f** *O. glabra* from Clade 12, **g** *O. purpurea* from Clade 7, **h** *O. flava* from Clade 8

Plant material was collected from the *Oxalis* research collection in the Stellenbosch University Botanical Gardens and supplemented with plant material collected in the field. The phylogenetic tree of Oberlander et al. (2011) [19] was used to guide taxon selection, to ensure even sampling across all of the main *Oxalis* clades (Fig. 1). Our sampling included 149 accessions (109 species) of native taxa and three non-native out-group accessions (three species). All accessions are living plants and each accession comprises of up to five individual plants. Accession numbers are reported as MO-numbers (see Additional file 1: Table S1), which are given in parentheses after species names throughout this study. A minimum of five mature leaves were sampled per accession, and in taxa with more than one leaflet, only central leaflets were studied. The datasets supporting the conclusions of this paper are included within the text (and its additional files).

### Leaflet conduplication

Many *Oxalis* taxa have conduplicate leaflets (AD leaflet halves folded along the central vein to face each other, with the fold facing the stem and the AB sides of the leaflets facing outwards). We coded leaflets as conduplicate or flat and sought to measure the degree of conduplication using the angles of the leaflets relative to the central vein. We collected all material during the day to control for potential nastic movements, and used both wax-embedded and fresh leaf material to verify leaflet conduplication.

### Epidermal traits

Epidermal impressions were made to study the AD and AB leaflet surfaces of mature leaves by applying clear nail varnish to fresh leaflet material. The nail varnish layers were peeled off with clear cellotape and stuck onto microscope slides for photography. Epidermal cell types were identified based on these epidermal impressions. The stomatal central axis length was measured as stomata are dynamic structures that can open and close, and this movement influences the stomatal short axis length [57].

### Wax-embedded and stained material

Fresh leaflet material was fixed in Formalin-Acetic-Acid, dehydrated in an alcohol series and gradually infiltrated with and embedded in paraffin wax [58]. A rotary microtome (Leitz, Germany) was used to cut transverse sections that ranged in thickness between 10 and 12 μm. Sections were stained using the Safranin-Alcian-blue [58] differential staining methods and DPX glue was used to preserve these sections as permanent slides.

### Data collection and measurements

Leaflet anatomical traits were studied using a Nikon ECLIPSE E400 light microscope and photographed using a Leica MC 170 HD camera and LAS CORE software (Leica, Switzerland). Digital images and permanent slides were used as reference material for comparative anatomical studies between the selected species. Images were edited in Microsoft Paint (Version 6.1) to clean the debris captured in the staining process from background of each image. Ten measurements of leaflet anatomical traits were taken in random fields of view for each studied leaflet trait, and the data collected from five leaflets were used to represent each accession. A total of 59 leaf anatomical traits representing dermal, ground and vascular tissue were recorded, of which 35 were qualitative (discrete) and 24 quantitative (continuous) (see Additional file 1: Table S1). Continuous traits were measured to scale from images imported to ImageJ [59]. To our knowledge, many of the discrete and continuous characters described in our study have not previously been assessed or described for *Oxalis* species.

### Phylogenetics

The 300 trees derived from the nuclear Internal Transcribed Spacer (ITS) region were used in this study, as ITS is more variable than plastid data and preliminary genome-level studies indicate that ITS-derived trees are more consistent with the species tree than plastid-derived phylogenies (K.C. Oberlander, unpublished data). The trees were reconstructed using an expanded ITS data set from Oberlander et al. (2011) [19], including sequences from a number of newly-collected taxa (see Additional file 2: Table S2) and extensive outgroup sampling of *Oxalis* and family Oxalidaceae. The basal resolution of the southern African clade in ITS-derived tree is very poor. Consequently we focus on the character states of 12 of the 13 clades identified by Oberlander et al. (2011) [19], as these clades receive either strong clade support in ITS-derived phylogenies, have palynological or other characters that support them, or both. As only 13 new taxa were included in the up-dated phylogeny, the trees are very similar to the original Oberlander et al. (2011) [19] tree as all of the numbered clades focal to this study are still extremely well-supported. The basal relationships using ITS in Clade 4 are still poorly supported, and despite looking different from Oberlander et al. (2011) in our featured tree, the posterior distributions are equally unresolved. The trees were generated in BEAST v.1.7.5 [60] using parameter settings for priors and Markov Chain Monte Carlo (MCMC) parameters as in Oberlander (2009) [22], with a normal prior of 56 million years (+/- 3.5 million years) on crown Oxalidaceae as described and motivated by Oberlander (2009) [22]. The taxa corresponding to Clade 7 were forced to monophyly, as many taxa in this clade have large deletions in ITS1 that negatively affect the resolution of this clade. However, both morphological characters [8] and preliminary genome-level data convincingly support these taxa as a monophyletic unit (unpublished data). Also, log likelihood and parameter values for unconstrained BEAST analyses were identical to clade-constrained analyses. Convergence of parameter values and the tree on the same posterior was checked in Tracer v.1.5. A burnin of the first 25 % of trees was removed, on a total of two independent runs of 1 X 10^7^ generations. All discrete and continuous traits were separately plotted on a sample of 10 phylogenetic trees chosen at random from the posterior distribution and one of these trees were chosen as a representative tree to plot all discrete and continuous data in this study. Where the same accession could not be sampled for both ITS and leaflet data, the tip MO-numbers on the tree were replaced by our accessions of the same species (see Additional file 1: Table S1). For ease of reference to the study of Oberlander et al. (2011) [19], we followed the same clade names and numbers in this study, except for the poorly characterised Clade 9, which is not retrieved in ITS data sets with greater taxon sampling and is not discussed further (Fig. 1). Only 6 discrete characters were considered to display a significant phylogenetic pattern, namely: AD epidermal cell types, AB epidermal cell types, stomatal position, mesophyll arrangement types, presence or absence of vascular sheaths and leaflet type. These traits were therefore regarded as the focal traits of this study.

### Ancestral state reconstruction

Ancestral states were reconstructed for the six above mentioned discrete leaflet anatomical traits, using maximum likelihood (ML) methods for 300 phylogenetic trees sampled from the BEAST posterior distribution [60]. The phylogenetic trees supporting the conclusions of this article are available in the TreeBase repository http://purl.org/phylo/treebase/phylows/study/TB2:S20073. All analyses were performed in R [61] using the ape [62] and geiger [63] packages, as well as custom scripts to remove tree tips without data. ML inferences were performed under default settings in ace from the ape package [62] in R [61]. To identify the best-fitting model of character evolution, the ER, SYM and ARD models implemented in the ace command were tested for each character across all 300 trees using the Akaike Information Criterion [64, 65]. In cases where the simpler model could not be rejected in favour of the more complex model, the simpler model was preferred. The model of best fit for each trait and tree was then used to reconstruct MCMC ancestral states for the 300 trees, using the default settings of simmap from the phytools package [66] in R [61]. The most likely character state at each node for Clades 1–13 was summed across all 300 trees (Table 1), and these are presented as pie charts at the relevant nodes in Fig. 5. Additionally, trait selection was verified by conducting ASR analyses for all other discrete characters. These analyses revealed high rates of evolution, confirming that the 6 traits identified had the most significant phylogenetic patterns (unpublished data).

**Table 1:** Ancestral character states estimated using MCMC models for six leaflet anatomical traits of *Oxalis*

	Clade1	Clade2	Clade3	Clade4	Clade5	Clade6	Clade7	Clade7b	Clade8	Clade9	Clade10	Clade11	Clade12	Clade13
Trait 1 (ER model)														
Irregular AD cells (crown)	**1.00**	**1.00**	**1.00**	**1.00**	**1.00**	**1.00**	**0.98**	0.01	**1.00**	**1.00**	**1.00**	**1.00**	**1.00**	**1.00**
Swollen AD cells (crown)	0.00	0.00	0.00	0.00	0.00	0.00	0.02	**0.99**	0.00	0.00	0.00	0.00	0.00	0.00
Irregular AD cells (stem)	**1.00**	**1.00**	**1.00**	**1.00**	**1.00**	**1.00**	**1.00**	0.01	**1.00**	**1.00**	**1.00**	**1.00**	**1.00**	**1.00**
Swollen AD cells (stem)	0.00	0.00	0.00	0.00	0.00	0.00	0.00	**0.99**	0.00	0.00	0.00	0.00	0.00	0.00
Trait 2 (ER model)														
Irregular AB cells (crown)	0.18	0.01	0.00	**0.98**	**1.00**	**1.00**	**0.98**	0.02	**1.00**	**1.00**	**1.00**	**1.00**	**1.00**	**1.00**
Swollen AB cells (crown)	0.00	0.00	0.00	0.00	0.00	0.00	0.02	**0.98**	0.00	0.00	0.00	0.00	0.00	0.00
Semi-swollen AB cells (crown)	0.82	**0.99**	**1.00**	0.02	0.00	0.00	0.00	0.00	0.00	0.00	0.00	0.00	0.00	0.00
Irregular AB cells (stem)	0.16	0.05	0.05	0.18	**1.00**	**0.99**	**1.00**	0.02	**1.00**	**1.00**	**1.00**	**1.00**	**1.00**	**1.00**
Swollen AB cells (stem)	0.00	0.00	0.00	0.00	0.00	0.00	0.00	**0.98**	0.00	0.00	0.00	0.00	0.00	0.00
Semi-swollen AB cells (stem)	0.83	**0.94**	**0.94**	0.82	0.00	0.01	0.00	0.00	0.00	0.00	0.00	0.00	0.00	0.00
Trait 3 (ER model)														
Epistomaty (crown)	0.01	0.00	0.00	0.70	0.54	**0.99**	**0.98**	0.01	**0.99**	**1.00**	**1.00**	**1.00**	**1.00**	**1.00**
Hypostomaty (crown)	**0.99**	**1.00**	**1.00**	0.30	0.46	0.01	0.00	0.00	0.01	0.00	0.00	0.00	0.00	0.00
Amphistomaty (crown)	0.00	0.00	0.00	0.00	0.00	0.00	0.02	**0.99**	0.00	0.00	0.00	0.00	0.00	0.00
Epistomaty (stem)	0.01	0.00	0.00	0.01	0.54	**0.99**	**0.99**	0.01	**0.99**	**1.00**	**1.00**	**1.00**	**1.00**	**1.00**
Hypostomaty (stem)	**0.99**	**1.00**	**1.00**	**0.99**	0.46	0.01	0.01	0.00	0.01	0.00	0.00	0.00	0.00	0.00
Amphistomaty (stem)	0.00	0.00	0.00	0.00	0.00	0.00	0.00	**0.99**	0.00	0.00	0.00	0.00	0.00	0.00
Trait 4 (SYM model - 82 %)														
Bifacial (crown)	**0.99**	**0.97**	**1.00**	**1.00**	**0.98**	**1.00**	**1.00**	**1.00**	**1.00**	0.85	0.56	0.00	0.00	0.00
Isobilateral (crown)	0.01	0.03	0.00	0.00	0.02	0.00	0.00	0.00	0.00	0.15	0.44	0.01	**1.00**	0.01
Homogenous (crown)	0.00	0.00	0.00	0.00	0.00	0.00	0.00	0.00	0.00	0.00	0.00	**0.99**	0.00	**0.99**
Bifacial (stem)	**0.97**	**0.99**	**0.99**	**0.99**	**0.98**	**1.00**	**1.00**	**1.00**	**1.00**	0.85	0.85	0.55	0.24	0.00
Isobilateral (stem)	0.03	0.01	0.01	0.01	0.02	0.00	0.00	0.00	0.00	0.15	0.15	0.45	0.76	0.01
Homogenous (stem)	0.00	0.00	0.00	0.00	0.00	0.00	0.00	0.00	0.00	0.00	0.00	0.00	0.00	**0.99**
Trait 4 (ARD model - 18 %)														
Bifacial (crown)	0.00	0.53	**1.00**	0.00	0.68	0.00	0.28	0.36	0.00	0.00	0.00	0.00	0.00	0.00
Isobilateral (crown)	0.00	0.15	0.00	0.00	0.21	0.42	0.45	0.49	0.51	0.02	0.00	0.00	**0.92**	0.00
Homogenous (crown)	**1.00**	0.32	0.00	**1.00**	0.11	0.58	0.26	0.15	0.49	**0.98**	**1.00**	**1.00**	0.08	**1.00**
Bifacial (stem)	0.00	0.13	0.13	0.00	0.68	0.00	0.00	0.36	0.00	0.00	0.00	0.00	0.00	0.00
Isobilateral (stem)	0.00	0.06	0.02	0.00	0.21	0.06	0.42	0.49	0.51	0.02	0.00	0.00	0.58	0.00
Homogenous (stem)	**1.00**	0.81	0.81	**1.00**	0.11	**0.94**	0.58	0.15	0.49	**0.98**	**1.00**	**1.00**	0.42	**1.00**
Trait 5 (ER model)														
Sheath absent (crown)	0.49	0.56	0.50	0.47	0.45	0.41	0.48	0.52	0.37	0.33	0.17	0.07	0.11	0.42
Sheath present (crown)	0.51	0.44	0.50	0.53	0.55	0.59	0.52	0.48	0.63	0.67	0.83	**0.93**	0.89	0.58
Sheath absent (stem)	0.49	0.51	0.51	0.49	0.45	0.44	0.41	0.52	0.37	0.33	0.33	0.16	0.11	0.42
Sheath present (stem)	0.51	0.49	0.49	0.51	0.55	0.56	0.59	0.48	0.63	0.67	0.67	0.84	0.89	0.58
Trait 6 (ARD model)														
Flat leaflet (crown)	0.00	0.65	0.00	0.00	0.81	0.00	0.74	**0.94**	0.00	0.00	0.00	0.00	0.00	0.00
Conduplicate leaflet (crown)	**1.00**	0.35	**1.00**	**1.00**	0.19	**1.00**	0.26	0.06	**1.00**	**1.00**	**1.00**	**1.00**	**1.00**	**1.00**
Flat leaflet (stem)	0.00	0.00	0.00	0.00	0.81	0.00	0.00	**0.94**	0.00	0.00	0.00	0.00	0.00	0.00
Conduplicate leaflet stem)	**1.00**	**1.00**	**1.00**	**1.00**	0.19	**1.00**	**1.00**	0.06	**1.00**	**1.00**	**1.00**	**1.00**	**1.00**	**1.00**

The ancestral character states for all six leaf anatomical traits (1 − 6) and 13 well-supported southern African *Oxalis* clades (Clades 1 − 13), with the proportion of trees with the reconstructed character state at that node across all 300 trees. Values greater than 90 % are indicated in bold font

## Results

### Epidermal pavement cells

Leaflet epidermal pavement cells displayed variable anticlinal and periclinal cell wall shapes. Three epidermal cell types were commonly observed and identified based on the anticlinal wall shape (as seen from above) and the periclinal wall shape (as seen from the side) of each cell; irregular, semi-swollen or swollen.

**Fig. 2:**
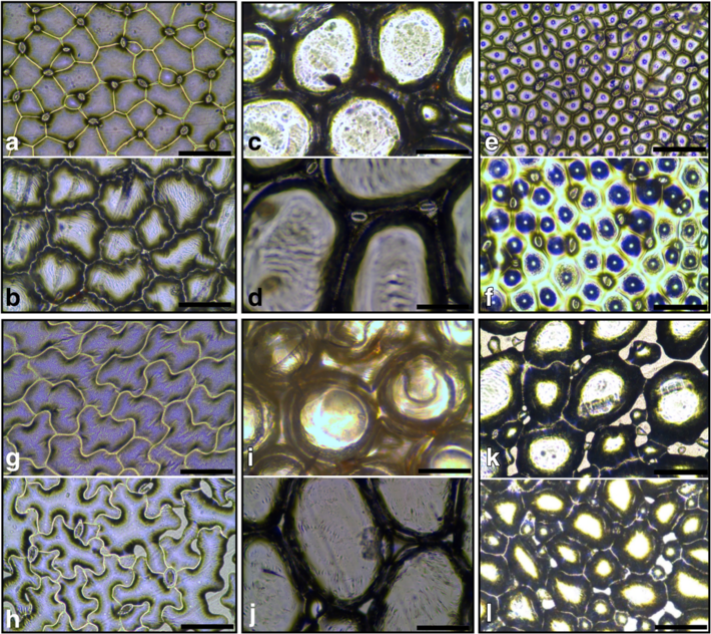
Light microscope photographs depicting different epidermal cell types observed in southern African *Oxalis* taxa. Irregular AD cells: **a** *O. purpurea* (MO344), **b** *O. heterophylla* (MO523), Swollen AD cells: **c** *O. pulchella* (MO559), **d** *O. nortieri* (MO503), Papillose AD irregular cells: **e** O*. inconspicua* (MO569), **f** *O. monophylla* (MO584), Irregular AB cells: **g** *O. uliginosa* (MO394), **h** *O. fenestrata* (MO1527), Swollen AB cells: **i** *O. foveolata* (MO1466), **j** *O. nortieri* (MO503), Semi-swollen AB cells: **k** *O. pes-caprae* (MO93), **l** *O. livida* (MO361). All scale bars represent 100 μm

**Fig. 3:**
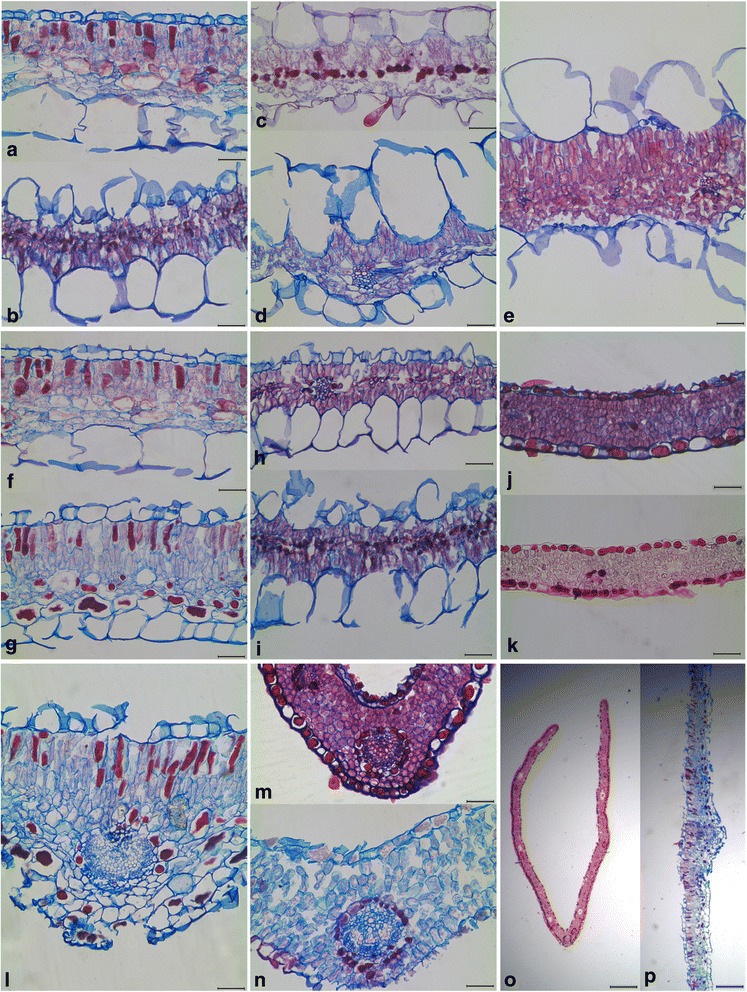
Light microscope photographs of cross sections through leaflet material, depicting various anatomical traits of *Oxalis*. Epistomaty: **a** *O. purpurea* (MO344), **b** *O. ericifolia* (MO1143), Hypostomaty: **c** *O. davyana* (MO1541), **d** *O. lateriflora* (MO887), Amphistomaty: **e** *O. foveolata* (MO1466), Bifacial mesophyll: **f** *O. purpurea* (MO344), **g** *O. melanosticta* (MO1485), Isobilateral mesophyll: **h** *O. cathara* (MO582), **i** *O. ericifolia* (MO1143), Homogenous mesophyll: **j** *O. tenella* (MO264), **k** *O. oreophila* (MO270), Sheath absent: **l** *O. melanosticta* (MO1485), Sheath present: **m** *O. oreophila* (MO270), **n** *O. ciliaris* (MO329), Leaflet conduplication: **o** *O. clavifolia* (MO556), **p** *O. melanosticta* (MO1485). Scale bars represent 100 μm, except for Trait 6 where scale bars represent 500 μm. All leaflets are orientated with the AD surface at the top of each image, and the AB surface at the bottom (except in (**p**) where the AD surface is to the left)

**Fig. 4:**
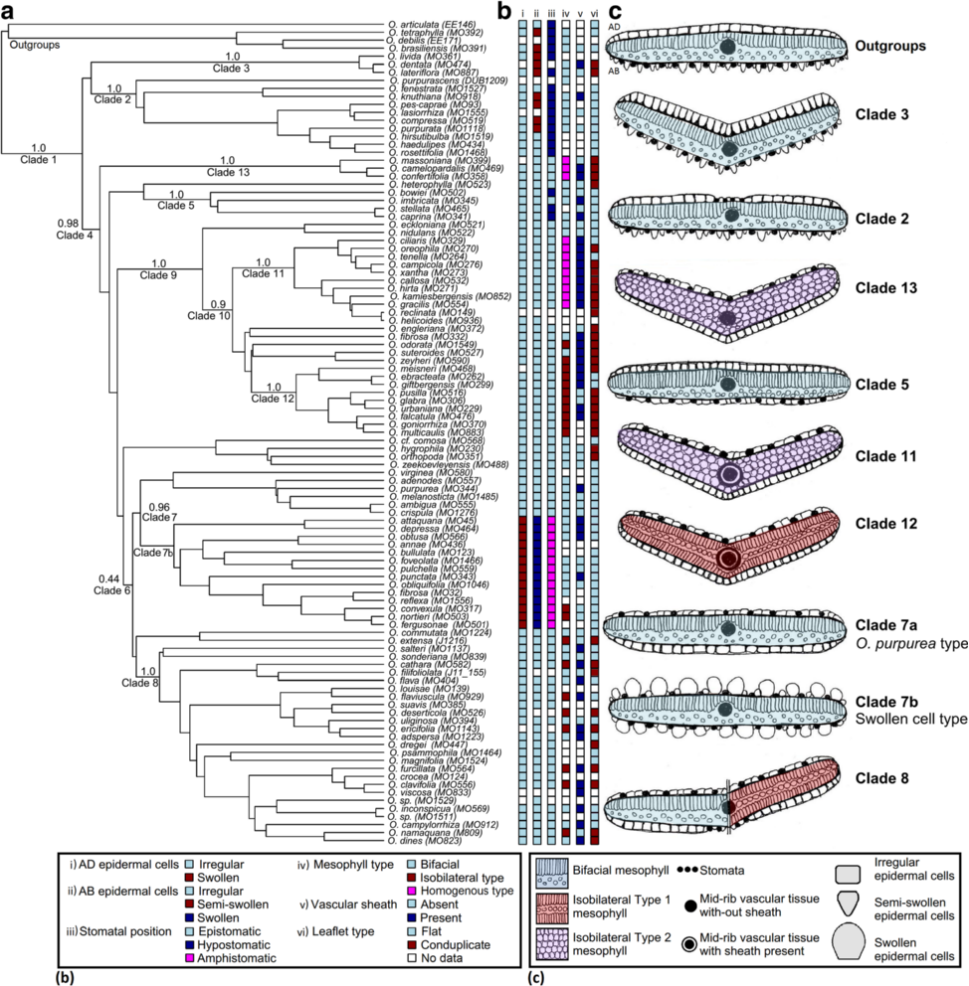
A phylogenetic tree with discrete-data and illustrations of 6 leaflet anatomical traits observed in *Oxalis*. **a** A single ITS phylogenetic tree from the BEAST posterior distribution for southern African *Oxalis* taxa. Posterior probability values for relevant clades are indicated above nodes. **b** The discrete data of six leaflet anatomical traits. **c** Illustrations representing the typical leaflet morphological and anatomical types of each of the numbered *Oxalis* clades. Leaflets orientated as in the outgroup example. AD – adaxial, AB – abaxial

Irregular epidermal cell types had anticlinal cell walls that varied in shape in a continuum from angular to sinuous (Fig. 2a, b, g, h). The periclinal walls were always parallel to the base of the epidermal cells (the point where the epidermal pavement cells and the guard cells of the stomata join), meaning that each irregular epidermal cell was uniform in depth. All irregular cells present on a leaflet surface appeared to be relatively similar in shape and size and this epidermal type occurred on either or both surfaces. Taxa with irregular AD and AB epidermal cells were scattered throughout the phylogeny (Fig. 4b i and b ii). Some irregular epidermal cells had a definite central conical protrusion in the outer periclinal wall of each cell, and these papillose epidermal cells were found only on the AD leaflet surface (Fig. 2e, f). Taxa with papillose type AD cells were scattered throughout the phylogeny (see Additional file 3: Figure S1b). The semi-swollen epidermal cell type was defined by the protruding outer periclinal wall tapering regularly towards a point above the centre of each AB epidermal cell, causing these cells to appear conical in shape (Fig. 2k, l). The anticlinal walls of these epidermal cells were angular to sinuous in shape. Conical-shaped cells were regularly interspersed by smaller and flatter epidermal cells and stomata and were only located on the AB surfaces of leaflets. Semi-swollen epidermal cell types were present in outgroup taxa as well as the species-poor deep-branching Clades 2 and 3 (Fig. 4b iii). The swollen epidermal cell type was defined by large, rounded cells that appeared to be swollen 3X-7X taller and 2X-5X wider than adjacent epidermal cells (Fig. 2c, d, i, j). When seen from above, the anticlinal cell walls varied from a rounded angular shape to spherical. Viewed in transverse section the periclinal wall was completely domed. The swollen cells were irregularly interspersed by small epidermal cells and stomata and were found on both the AD and AB leaflet surfaces. Swollen epidermal cells were observed only in Clade 7b (14 out of 14 taxa) rendering this trait unique to this sub-clade (Fig. 4b i and Fig. 3b ii).

### Stomata

Unexpectedly, three stomatal positions were observed; epistomatic (Fig. 3a, b) taxa were observed in 70 out of 110 taxa (63.6 %) and were more common than hypostomatic (Fig. 3c, d) taxa (14.5 %) and amphistomatic (Fig. 3e) taxa (21.8 %). Stomatal position showed a strong phylogenetic pattern as all outgroup taxa and all taxa from Clades 2 and 3 had hypostomatic leaflets. In contrast, all but three taxa from the large Clade 4 had epistomatic leaflets, with three exceptions confined to Clade 5. Within Clade 4, Clade 7b was the only clade with amphistomatic leaflets (14/14 species) (Fig. 4b iii).

Interestingly, 32 taxa appeared to have the majority of their stomata on one leaflet surface (i.e. epistomatic or hypostomatic), but with a few additional stomata on the opposite leaflet surface above the central vein. Thirteen of these taxa were predominately epistomatic and have conduplicate leaflets, with a few additional vein-associated stomata on the AB leaf surface. Seven taxa were predominately hypostomatic and had flat leaflets, but with additional stomata on the AD leaflet surface above the central vein. The remaining 12 taxa (with additional stomata on leaflet surfaces) did not display the two previously described combinations of stomatal position and leaflet conduplication. All observed amphistomatic taxa had bifacial mesophyll arrangement and flat leaflets. Epistomatic taxa had both bifacial and isobilateral mesophyll arrangement in both flat and conduplicate leaflets. Hypostomatic taxa had bifacial mesophyll arrangement and leaflets were always flat. Stomata of all taxa appeared to be very slightly sunken below the plane where epidermal pavement cells and the first layer of mesophyll cells meet, regardless of the size or shape of the epidermal and mesophyll cells.

Four types of stomatal complexes were observed in *Oxalis* (definitions based Radford et al. 1974 (2)), namely anomocytic, anisocytic, actinocytic and an unusual 4-celled anisocytic stomatal type (comprising of three large cells and one small cell). All four types of stomatal complexes were commonly encountered in different combinations, frequently on the same leaflet, and did not show any clear phylogenetic patterns (see Additional file 3: Figure S1d, e, f, g). No obvious phylogenetic patterns of continuous stomatal traits, such as large increases or decreases of stomatal length or density confined to specific clades, were detectable in any of the *Oxalis* clades (data not shown).

### Trichomes

Two main types of trichomes were observed among *Oxalis* taxa, namely glandular- or non-glandular hairs, depending on the presence or absence of swollen secretory cells at the tip of the trichome. Two subtypes of glandular and five subtypes of non-glandular hairs were recognised. Non-glandular hairs were more common (78.7 % across taxa) than glandular hairs. The leaflet epidermal surfaces of only 29 out of 110 studied *Oxalis* species (26.4 %) were glabrous, i.e. completely free of trichomes, while the other taxa had at least one type of trichome present. Trichomes were most commonly observed on the AB leaflet surface (54.5 % across taxa). Taxa with trichomes on both surfaces were less common (18.2 %) and only one species (*O. foveolata* MO1466) had trichomes solely present on the AD leaflet surface. Considerable variation of trichome traits was observed between individuals from the same species and between garden and field-collected samples, implying considerable intraspecific variability and little phylogenetic pattern (see Additional file 3: Figure S1i, j, k). The margins of leaflets were observed to be glabrous, simple hair-ciliate (non-glandular hairs) or glandular hair-ciliate. These ciliation types were equally common, as a third of the studied taxa displayed each of the described ciliation-types. The ciliation types did not show any large-scale pattern, but small clusters of taxa (three to five closely related species) within a clade often shared ciliation types throughout the *Oxalis* phylogeny (see Additional file 3: Figure S1l).

### Mesophyll arrangement

Mesophyll arrangement types were consistent between multiple samples of the same *Oxalis* species. Three mesophyll arrangement types were observed in this study, depending on the relative arrangements of, and differentiation between, tissue consisting of cylindrical palisade cells and tissue consisting of round/oval spongy mesophyll cells. The majority of studied taxa (55.2 %) had bifacial mesophyll i.e. clear differentiation between an AD palisade parenchyma and AB spongy parenchyma (Fig. 3f, g). Two different types of mesophyll arrangements were observed in the remaining taxa: isobilateral mesophyll was defined as an arrangement with usually one (and up-to three) layers of round-celled spongy parenchyma located between upper and lower palisade layers (Fig. 3h, i). The spongy mesophyll tissue was arranged in the same plane as the vascular tissue and the AD and AB palisade layers had approximately the same thickness. Homogenous mesophyll was defined as mesophyll arrangement where only uniform oval-shaped parenchyma cells occur in the mesophyll tissue, and distinct palisade and spongy mesophyll layers could not be distinguished (Fig. 3j, k). Isobilateral mesophyll (26 out of 39 taxa) was more common than homogenous mesophyll.

The mesophyll of bifacial taxa was differentiated into one to five-seriate palisade parenchyma and two to six-seriate spongy parenchyma. The palisade parenchyma in bifacial arrangements usually comprised slightly over half (51.2 %, average = 59.4 μm, range = 24.9 μm - 178.9 μm) of total mesophyll thickness (spongy parenchyma average = 55.7 μm, range = 19.8 μm - 88.4 μm). The mesophyll of isobilateral taxa was differentiated into an AD and AB layer of one to three-seriate palisade parenchyma each, and one to three-seriate spongy parenchyma located between the palisade layers. Palisade parenchyma comprised almost two thirds (63.2 %, average = 72.3 μm, range = 35.6 μm - 186.0 μm) of total mesophyll thickness (spongy parenchyma average = 25.4 μm, range = 9.5 μm - 61.0 μm) in leaves of this type. The mesophyll of homogenous taxa was not differentiated into a palisade and spongy layer, but seemed to match only spongy parenchyma (cells round to ovoid). The average mesophyll heights ranged from 58.9 μm to 158.0 μm.

All sampled outgroup taxa, Clade 2, Clade 3, Clade 5 and Clade 7 (except *O. nortieri* (MO503) and *O. dilatata* (MO524)) had bifacial mesophyll arrangement types. Taxa from Clade 12 had isobilateral arrangement and taxa from Clade 8 had both bifacial and isobilateral arrangements. Taxa from Clade 11 and Clade 13 had homogenous mesophyll arrangement. Mesophyll type was therefore a trait with a relatively clear phylogenetic pattern (Fig. 4b iv).

### Vascular tissue

Two types of venation were identified within studied leaflets, namely pinnate (typical central vein and secondary veins branching from the central vein, usually opposite each other) and palmate venation (veins branch from 1 single point, close to the leaflet articulation point). Taxa with palmate venation occurred only in Clade 7 (6 taxa) and Clade 8 (2 taxa) (see Additional file 3: Figure S1q). As in standard angiosperm leaf vascular tissue, the midrib vascular trace of all studied taxa contained xylem that faced the AD surface and phloem that faced the AB surface [67]. The vascular bundles were located at the junction of palisade and spongy mesophyll in all taxa with bifacial mesophyll arrangement, and in the middle region of the mesophyll of all isobilateral taxa. The central and lateral vascular bundles of 48 out of 84 studied taxa (57.1 %) were surrounded by a single layered sheath that was different in size and shape to the surrounding parenchyma cells in the mesophyll. Transverse sections of these cells revealed that they were circular or ovate in shape, and the cell walls and contents stained differently to the surrounding parenchyma cells (Fig. 3m, n). The presence or absence (Fig. 3l) of a sheath around vascular tissue was constant throughout samples of the same species. Taxa with a sheath around vascular tissue were distributed throughout the phylogeny, but showed some phylogenetic pattern as the majority of taxa from Clade 10 had the sheath around their vascular tissue (Fig. 4b v). The vascular tissue and mid-rib region of 38 out of 89 studied taxa (42.4 %) projected outwards on the AB surface, while the remainder of taxa had vascular tissue embedded in the mesophyll. The presence or absence of a protruding mid-rib was constant throughout samples of the same species, but this trait was not unique to any *Oxalis* clades (see Additional file 3: Figure S1p).

### Leaflet conduplication

Flat (Fig. 3o) and conduplicate (Fig. 3p) leaflets were observed to be equally common throughout the studied taxa (50.0 % each), but did show some phylogenetic pattern (Fig. 4b vi). Flat leaflets had angles ranging from 111.5° to 180.0°, while conduplicate leaflets had angles ranging from 40.6° to 139.7°. This overlap between the angles of the flat and conduplicate leaflets is due primarily to locally increased folding around the central vein. Flat leaflets were observed in all members of the outgroup taxa, Clade 2, Clade 5 and Clade 7 and members from Clades 3, 11, 12 and 13 had conduplicate leaflets. Clade 8 had a mixture of both flat and conduplicate leaflets.

*Oxalis* taxa have variable numbers of leaflets per leaf, ranging from one, to three (89.2 % of studied taxa) up to 29 [8], but with a maximum of eleven leaflets observed in this study. Taxa with three leaflets were by far the most commonly observed, and were distributed throughout the *Oxalis* phylogeny. Taxa with more than three leaflets per leaf were found within four clades (outgroup taxa, Clades 8, 10 and 12) and taxa with single leaflets were located in two clades (Clades 7 and 8) (see Additional file 3: Figure S1a).

### Summary of Oberlander et al. (2011) [19] clades with unique combinations of traits

All sampled outgroup taxa were characterised by the combination of having flat leaflets, AB located stomata, bifacial mesophyll arrangement, vascular tissue without a sheath and semi-swollen epidermal cells on the AB leaflet surface (except *O. articulata* (EE146) with irregular cells).

#### Clade 1 - South African *Oxalis*

No phylogenetically significant traits were observed that delineate this clade. However, because our focus was on potential synapomorphic characters within the southern African radiation, only four closely related outgroup taxa were sampled in our study, hindering our ability to detect characters unique to and universal to this clade. Given the strong support for monophyly and very distinctive bulb types of this clade, it is quite possible that leaflet anatomical synapomorphies do exist. Potential characters include some of the new stomatal complex types found in this study, but these require greater outgroup sampling to confirm.

#### Clade 2 - *O. pes-caprae* and relatives

All taxa from Clade 2 had flat leaflets. Stomata were always located on the AB leaflet surfaces and additional stomata were located on the AD leaflet surfaces above the mid-rib of all taxa (except *O. hirsutibulba* (MO1519), which was hypostomatic without additional AD stomata). Semi-swollen epidermal cells were located on the AB leaflet surfaces of most taxa from this clade. Mesophyll arrangement of all taxa was bifacial and vascular tissue was without a sheath.

#### Clade 3 - sect. *Cernuae* subsect. Lividae

Taxa from Clade 3 had conduplicate leaflets. The AD epidermal cells were exceptionally thick. Semi-swollen epidermal cells were located on the AB leaflet surfaces in combination with AB located stomata. Mesophyll arrangement was always bifacial and vascular tissue was without a sheath.

#### Clade 4 - Core South African *Oxalis*

AD located stomata is a diagnostic trait for the majority of this species-rich southern African *Oxalis* clade (except for Clade 5 with hypostomatic leaflets).

#### Clade 5 - *O. stellata* and relatives

All taxa from Clade 5 had flat leaflets with bifacial mesophyll arrangement. Leaflets were hypostomatic (except for one epistomatic taxon). All taxa from this clade had vascular tissue without a sheath. Leaflets were ciliated with non-glandular hairs.

#### Clade 7a - *O. purpurea* and relatives

The majority of taxa had flat leaflets with bifacial mesophyll arrangement. All leaflets had AD located stomata and vascular tissue was without a sheath. The majority of species from this sub-clade were glabrous on both leaflet surfaces and the majority of leaflets were not ciliated. Palmate venation was very common among taxa from this sub-clade.

#### Clade 7b - *O. purpurea* and relatives

Taxa had flat leaflets with bifacial mesophyll arrangement and vascular tissue was without a sheath. Swollen epidermal cells and stomata on both the AD and AB leaflet surfaces were synapomorphic traits of this sub-clade. Mesophyll with cavities (with and without epithelial lining) was common in this clade. Many species had non-glandular hairs and/or glandular hairs on the AB or both leaflet surfaces.

#### Clade 8 - *O. flava* and relatives

Leaflets were epistomatic and additional stomata located on the AB main vein surface were very common in this clade. These additional stomata were located in between elongated AB epidermal cells. Leaflets were either flat or conduplicate with bifacial or isobilateral mesophyll arrangements. Many of taxa from this clade had glabrous leaflets. If taxa were not glabrous, glandular hairs (long- and short-stalked) were commonly observed (this is one of only two clades with glandular hairs). Bifacial or isobilateral mesophyll types were observed and vascular tissue was without a sheath. The majority of species with less than three leaflets per leaf (3/5 species) and more than three leaflets per leaf (5/8 species) belong to this clade. Some species from this clade with ciliated leaflets had glandular-haired cilia (this is one of the two southern African *Oxalis* clades with glandular haired cilia (long- or short-stalked) on leaflets).

#### Clade 10

Leaflets from this clade had isobilateral and homogenous mesophyll arrangements (except for two taxa with bifacial mesophyll arrangement). A sheath was present around the vascular tissue in all but one taxon from this clade. All leaflets were epistomatic and conduplicate.

#### Clade 11 - *O. hirta* and relatives

All taxa from Clade 11 had conduplicate leaflets. Stomata were always located on the AD leaflet surfaces (epistomatic) and mesophyll arrangement was always homogenous. Sheaths around the vascular tissue were observed in all taxa from this clade. 80 % of taxa had AB located trichomes.

#### Clade 12 - *O. glabra* and relatives

All taxa from Clade 12 had conduplicate leaflets with isobilateral mesophyll arrangements. All leaflets were epistomatic and had sheaths around the vascular tissue. Glandular hairs were commonly observed (this is one of only two clades with glandular hairs (short stalked)). If trichomes were present, they were located only on the AB leaflet surfaces.

#### Clade 13 - Sect. *Angustatae* subsect. *Pardales*

All taxa from Clade 13 had conduplicate leaflets with homogenous mesophyll arrangements. All leaflets were epistomatic, had cavities with epithelial linings in their mesophyll and vascular tissue was without a sheath. Trichomes (non-glandular and glandular hairs) were located on the AB surface only.

### Ancestral state reconstruction

AIC values universally chose the ER model for Traits 1–3 and 5, ARD for Trait 6, and strongly favoured SYM for 82 % of trees for Trait 4 (remaining 18 % of trees were better fit with the ARD model). We considered a character state confidently inferred when >90 % proportion of trees had the same most likely character state at a node (Table 1). Although this does not measure the direct support of competing character states at this node in each tree, we consider it a useful proxy of this value, accounting for the great variability in the ITS tree topology. The ranges of character rate change across all 300 trees are presented in Table 2. Assignment of a state change to a particular branch was considered strong when the stem and crown nodes were confidently reconstructed as differing character states.

**Table 2:** The rates of ancestral character state evolution for six leaflet anatomical traits of *Oxalis*

	0 to 1	0 to 2	1 to 0	1 to 2	2 to 0	2 to 1
AD epidermal cell types (ER model)	0.002 (0.001 - 0.004)		0.002 (0.001 - 0.004)			
AB epidermal cell types (ER model)	0.004 (0.003 - 0.007)	0.004 (0.003 - 0.007)	0.004 (0.003 - 0.007)	0.004 (0.003 - 0.007)	0.004 (0.003 - 0.007)	0.004 (0.003 - 0.007)
Stomatal position (ER model)	0.004 (0.002 - 0.006)	0.004 (0.002 - 0.006)	0.004 (0.002 - 0.006)	0.004 (0.002 - 0.006)	0.004 (0.002 - 0.006)	0.004 (0.002 - 0.006)
Mesophyll type (SYM model - 82 %)	0.050 (0.032 - 0.073)	0.004 (0 - 0.008)	0.050 (0.032 - 0.073)	0.008 (0 - 0.025)	0.004 (0 - 0.008)	0.008 (0 - 0.025)
Mesophyll type (ARD model - 18 %)	0.011 (0 - 0.037)	0 (0 - 0.007)	0.145 (0.096 - 0.208)	0.001 (0 - 0.015)	0.061 (0 -0.122)	0.126 (0 - 0.210)
Vascular sheath (ER model)	0.117 (0.071 - 0.188)		0.117 (0.071 - 0.188)			
Leaflet type (ARD model)	0.000		0.096 (0.059 - 0.151)			

**Fig. 5:**
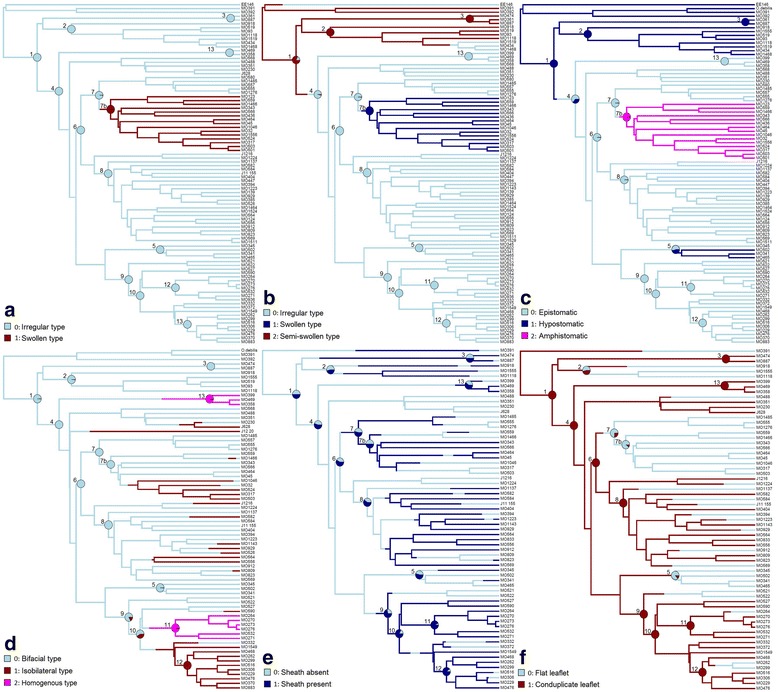
Ancestral state reconstruction analysis output plotted onto a phylogenetic tree for southern African *Oxalis*. All ancestral state reconstruction analyses made used MCMC models for the following traits: AD epidermal cell types (**a**), AB epidermal cell types (**b**), stomatal position (**c**), mesophyll arrangement typse (**d**), vascular sheath (**e**) and leaflet conduplication (**f**). Trees look different due to missing values for some tip taxa. Pie charts present the proportion of most likely character states across a sample of 300 trees for the focal 13 Clades of this study (as numbered from 1 to 13 on each phylogeny). Only accession numbers are plotted (in order to optimize space), but correspond to the species names as plotted in Fig. 4

The range of rates of ancestral character state evolution estimated using MCMC models for 6 leaflet anatomical traits of southern African *Oxalis*. Average, minimum and maximum (min - max) range values are reported for models run across all 300 trees. For character states (states 0, 1 and 2) see figure legends in Fig. 5

**Trait 1** - AD epidermal cell types (Fig. 5a): The irregular AD epidermal cell type is ancestral to all well supported southern African *Oxalis* clades, except in Clade 7b with the swollen AD epidermal cell type. There is clear support to show that the switch between these two character states took place on the branch subtending Clade 7b (as this trait is not present in the stem node i.e. crown Clade 7).

**Trait 2** - AB epidermal cell types (Fig. 5b): The ancestral state to all southern African *Oxalis* is equivocal (82 % of trees with semi-swollen AB cells). The reconstructed state for Clades 2 and 3 are semi-swollen AB epidermal cells, which agrees with the distribution of this character in these clades. The ancestral state to Clade 4 is reconstructed as the irregular cell type, and the switch from semi-swollen to irregular cell types was reconstructed on the branch subtending Clade 4. The irregular epidermal cell type is the well-supported ancestral state to all of the sampled Oberlander et al. (2011) [19] clades (except Clade 7b) within Clade 4. The swollen epidermal cell type evolved once in the southern African *Oxalis* phylogeny, and this is the ancestral character state to Clade 7b.

**Trait 3** - Stomatal position (Fig. 5c): Hypostomatic leaflets are reconstructed as the ancestral state to all southern African *Oxalis*, as this trait is present in the outgroup samples and there is clear support to indicate that this is the ancestral states to Clades 1, 2 and 3. All of the well-supported clades within Clade 4 (except Clade 5 with an equivocal epistomatic or hypostomatic ancestral state) have ancestral states of epistomatic leaflets, while the ancestral state to Clade 4 is equivocal (with strong support for the presence of hypostomaty at the stem to Clade 4). This is most likely due to the relatively uncertain placement of Clade 5 within this set of *Oxalis* phylogenies. The change from epistomatic to amphistomatic leaflets is unequivocally reconstructed as occurring on the branch subtending Clade 7b.

**Trait 4** - Mesophyll arrangement types (Fig. 5d): For the majority of trees, the SYM model was preferred and a bifacial mesophyll arrangement was the ancestral state to the majority of the southern African *Oxalis* clades (as seen in Clades 1 to Clades 8). Isobilateral mesophyll evolved multiple times across the phylogeny, and this trait appears to be the well-supported ancestral state to Clade 12, while the ancestral state at the stem of this clade is equivocal. Homogenous mesophyll arrangements evolved twice in this *Oxalis* phylogeny and this trait is the well-supported ancestral state to Clades 11 and 13. The outcome of trees that preferred the ARD model is very different: isobilateral mesophyll is still the well-supported ancestral state to Clade 12, bifacial mesophyll is reconstructed here as a derived state, and homogenous mesophyll is the ancestral state to southern African *Oxalis* and the majority of southern African *Oxalis* clades. However, the ARD model is only the best-fitted model for 18 % of all trees. In these trees the ARD model reconstructs high rates of change away from the uncommon homogenous state (Table 2). Such counterintuitive behaviour of more complex models in ancestral state reconstruction where one character state is rare (such as homogenous mesophyll here), has long been known [68]. Although the ARD model fit this subset of trees substantially better than the SYM model, we argue that the chances of homogenous mesophyll being ancestral in SA *Oxalis* are unlikely, given the distribution of bifacial mesophyll in other southern African *Oxalis* taxa (as 55.2 % of studied taxa had bifacial mesophyll while only 14.9 % of taxa had homogenous mesophyll) and bifacial mesophyll in some studied outgroup taxa. We could not find any major differences in total tree length or in tree depth (age) between these two groups of trees, nor were there any obvious topological differences (data not shown). Interestingly, in both models we observed directionality to mesophyll type evolution. Change between bifacial and homogenous mesophyll tended to occur through an isobilateral intermediate (Table 2) – rates between bifacial and homogenous mesophyll were generally the lowest across both models and all 300 trees. Although preliminary, this has possible functional implications for the evolution of isobilateral mesophyll.

**Trait 5** - Vascular sheath (Fig. 5e): The ancestral condition to the majority of the *Oxalis* clades was equivocal. The scattered distribution of this character across the tree resulted in high reconstructed rates of change, leading to uncertainty at deeper nodes. The estimated rates of change between character states were considered as being high in comparison to the previous four traits (Table 2), and possibly indicate that the presence or absence of a vascular sheath is a trait that is easily gained or lost and therefore has less phylogenetic significance than originally assumed. Despite this, the ancestor of Clade 11 has strong support for the presence of a vascular sheath and this character is therefore systematically useful for this clade.

**Trait 6** - Leaflet conduplication (Fig. 5f): The reconstructed ancestral condition to Clades 1, 3, 4, 6, 8, 9, 10, 11, 12 and 13 was strongly supported as conduplicate leaflets. The ancestral state to the remainder of clades was equivocal. However, flat leaflets were reconstructed as the ancestral state to Clade 7b, and there is clear support to indicate that the switch between traits took place on the branches leading to this clade.

## Discussion

### Epidermal pavement cells

Various comparative leaf anatomical studies include epidermal pavement cell types [40–42] and refer to cells with straight or sinuous anticlinal walls as two separate cell types. In southern African *Oxalis* both typical sinuous and angular anticlinal cell walls were observed. However, these two states were extremes in a continuum of tremendous variability, which proved difficult to categorise, sometimes varying even within individuals of the same species. The variability of this trait might be better explained as a response to environmental factors. The pavement cells of plants growing in shade reportedly have more sinuous anticlinal walls than plants exposed to full sun, which might very well explain the variation we observed [69–71]. Irregular epidermal cell types occurred in all examined species and on both leaflet surfaces and are reconstructed as the ancestral state for the AD leaflet surface in southern African *Oxalis*. Similarly, although a papillose AD epidermis was very common in our sampled taxa, we could find no phylogenetic pattern in the distribution of this trait and it is possible that it too is environmentally-induced.

The semi-swollen epidermal cell type was confined to the outgroup taxa, Clade 2 and Clade 3, but not all taxa from these clades had this epidermal cell type. The AB epidermal cells of sect. *Ionoxalis* are described as having large epidermal cells relative to the width of the leaflets [13], and agrees with Metcalfe and Chalk’s (1950) [72] description of “arched” cells (convex) that resemble palisade cells. ‘Colliculate’ AB epidermal cells have been recorded in hypostomatic leaves of Chilean *Oxalis* species, and these cell types appear to be similar to our semi-swollen cell types recorded in the hypostomatic *Ionoxalis* and Clades 2 and 3 [73]. The presence of semi-swollen AB epidermal cells in the outgroup taxa and two (possibly three) of the deepest diverging clades in southern African *Oxalis* (Clade 2 and Clade 3 - possibly Clade 1) and the results of the ancestral state reconstruction show that the presence of semi-swollen cells on the AB epidermis is the ancestral state in southern African *Oxalis* Clades 2 and 3, with this cell type being lost in the ancestor to Clade 4.

Swollen epidermal cells found on both AD and AB surfaces of leaflets were unique to Clade 7b and appear to be the well-supported ancestral states to this clade as well, making this trait phylogenetically informative. Swollen epidermal cells have been described in southern African *Oxalis* [8] and on the AB surface of South American *O. carnosa* [47]. These swollen cells were described by Salter (1944) [8] as being “comparatively large” and the majority of swollen-celled *Oxalis* species were grouped into Salter’s (1944) [8] section *Foveolatae* with large epidermal cell size as one of the defining characteristics of this section. Salter’s (1944) [8] *Foveolatae* was almost identical to our Clade 7b, except that he included *O. furcillata*, which does not display the swollen epidermal cell type and is included in Clade 8. Salter (1944) [8] excluded *O. obtusa* from sect. *Foveolatae*, but both the ITS tree and the leaflet anatomical results indicate that *O. obtusa* does belong in Clade 7. Oberlander (2009) [22] suggested that this trait could be a synapomorphy for Clade 7, but our results show that this character state does not typify the entire clade, and would be more correctly considered a synapomorphy for Clade 7b. The currently un-sampled members of Section *Foveolatae* (*O. oreithala*, *O. algoensis* Eck. & Zey., *O. fourcadei* Salter, *O. lawsonii* Bol. and *O. senecta* Salter) are all listed as having the swollen epidermal cell type and consequently should also belong in Clade 7b. Examples from the literature have suggested that swollen epidermal cells of some *Oxalis* species work as lenses (lens mechanism) to focus light through the epidermis into the chloroplast-rich palisade cells [74–76]. Authors suggested that the increased light in the palisade tissue increases the photosynthetic performance of leaves, and it is possible that the swollen epidermal cell type found in southern African *Oxalis* could fulfil a similar function.

### Stomatal position

Hypostomaty is present in southern African *Oxalis*, which is the stomatal position consistently reported in the Oxalidaceae literature. A recent comparative study on three South American *Oxalis* species also reported all studied species to have AB located stomata (*O. latifolia* H.B.K., *O. debilis* Knuth, *O. corniculata* L.) [49]. Denton (1973) [13] found that North American species from section *Ionoxalis* had hypostomatic leaflets, which agrees with our findings in our sampled members of this section. AB-located stomata have also been described for *O. carnosa* [47]. *O. corniculata*, *O. carnosa* and members of section *Ionoxalis* are considered distantly related [9], so this information together with our ancestral state reconstruction analysis are consistent with the hypothesis that AB-located stomata are widespread and ancestral in *Oxalis*. Ancestral state reconstruction unambiguously retrieved AB-located stomata in the most recent common ancestor of southern African *Oxalis*. AB-located stomata also characterise the species-poor Clades 2 and 3, which have retained this ancestral character state according to our reconstructions.

Unexpectedly, the vast majority of southern African *Oxalis*, corresponding to the large radiation of Clade 4, are characterised by having stomata on the AD leaflet surface. The major exception to AD stomata in the speciose Clade 4 is in Clade 5, the *Stellata* Clade of Oberlander et al. (2011) [19], which apart from a single species *O. imbricata* (MO345)) is uniformly hypostomatic. This implies a fairly complex history of character change for stomatal position in the lineage leading towards Clade 5, which is consistent with the results from our ancestral state reconstructions. However, it must be noted that the *Stellata* Clade was one of the most unexpected clades retrieved by Oberlander et al. (2011) [19], comprising a mix of morphologically dissimilar species. Recent phylogenetic results with massively increased sampling from hundreds of low-copy nuclear loci cause this clade to disintegrate, with Clade 5 generally forming one branch of the basal bifurcation in Clade 4 and *O. imbricata* deeply embedded within the other branch with strong support (R. Schmickl, unpublished data). These more recent results are more consistent with the morphology of the species involved [8] and imply a much simpler scenario for the evolution of stomatal position deep in the southern African *Oxalis* lineage, with a single, un-reversed change from an ancestral hypostomatic southern African taxon to epistomatic leaflets within Clade 4.

Taxa with amphistomatic leaflets were observed only in Clade 7b and this is the well-supported ancestral state to this clade. Amphistomatic leaves frequently occur in xeric habitats [77] or strongly seasonal environments [78] and amphistomaty is regarded as an adaptation to enable the maximum conductance of a leaf [73, 79]. This is consistent with the highly seasonal nature of the GCFR climate, which might favour maximal stomatal conductance during winter periods when water is not limiting, providing amphistomatic taxa with a photosynthetic advantage [80]. It is interesting that all taxa with amphistomatic leaflets had swollen epidermal cell types on both the AD and AB leaflet surfaces. A similar association was reported in the Aizoaceae [81].

The presence of additional stomata above the mid-rib on either the AD and AB leaflet surfaces was not a trait with a strong enough phylogenetic pattern to typify clades, but frequently occurred in two clades (additional stomata on AD surface of Clade 2 and additional stomata on the AB surface of Clade 8). This trait was not observed in any of the outgroup taxa studied, nor mentioned in the literature on southern African or global *Oxalis* species, although the “amphistomatous” leaflets of *O. latifolia* in dos Reis and Alvim (2013) [49] might actually refer to this character state. This phenomenon has been recorded in other angiosperms, for example *Solanum* L. [40]. The spacing of stomata is theoretically not random [80]. Our data supports this and given the energetic cost of stomatal formation we suggest that these additional stomata above the midrib might have functional significance.

The presence of additional stomata above the central vein of leaflets is common among members from Clades 2 and 8, but scattered otherwise throughout the southern African *Oxalis* phylogeny. This suggests that this state can easily evolve in *Oxalis* species, but it does not persist for a long time. However, it does suggest a pathway for the change from ancestrally hypostomatic to derived epistomatic leaflets, through viable, short-lived intermediate stages, starting off with the gain of AD vein-associated stomata and their spread across the AD leaflet surface into *bona fide* amphistomaty. This, however, raises the interesting question as to why this process did not end with amphistomaty, but culminated in epistomatic leaflets in Clade 4. Epistomaty in the rarest stomatal position among angiosperms and the one considered to have the greatest evolutionary costs [80]. Yet the huge size discrepancy between the hypostomatic Clades 2 and 3, and the huge epistomatic Clade 4, suggest that epistomaty might have significant, as yet undetermined fitness benefits. It is possible that AD located stomata could serve as a key innovation for the massive Clade 4, which contains the vast majority of southern African *Oxalis* diversity.

### Mesophyll arrangement type

The mesophyll arrangement types observed in southern African *Oxalis* appeared to be phylogenetically informative traits. Bifacial mesophyll arrangement was the most common type among southern African *Oxalis*, and our analyses showed that this was the ancestral state to the majority of well-supported southern African clades. The presence of homogenous mesophyll arrangement is a trait that is present universally and ancestrally in two phylogenetically distant clades (Clade 11 (9/9 species) and Clade 13 (3/3 species)). Our analyses showed that this character state evolved twice in southern African *Oxalis*. To our knowledge there is no available morphological [8], palynological [21] or genetic [19] evidence to support a close relationship between these two clades. The isobilateral mesophyll arrangement evolved multiple times among southern African clades, and was the well-supported ancestral state to one *Oxalis* clade (Clade 12). Our ancestral character state analysis showed that some taxa from Clade 8 reverted between bifacial and isobilateral mesophyll arrangements, indicating possibly that this clade has an elevated rate of evolution of this character. Isobilateral and homogenous mesophyll arrangements are commonly observed in southern African *Oxalis* species. Many *Oxalis* species hold their leaves erect and away from the soil surface, with both leaflet surfaces potentially exposed to sunlight at different times of the day. Possibly isobilateral and homogenous mesophyll arrangements would have selective advantage under these circumstances, although this remains to be tested. The degree of conduplication also would have an effect on exposure to sunlight, and it is notable that almost all isobilateral taxa in our study have conduplicate leaflets. Neither argument can account for the lack of palisade in homogenous taxa, however, which must have a different explanation. There are some functional explanations in the literature that report changes from bifacial to isobilateral (and in some cases homogenous) mesophyll. A study on the evolution of Amarylidaceae suggest that this change can happen as a lineage moves into and becomes adapted to drier regions [82], as might have happened in the southern African O*xalis* lineages. An interpretation provided suggested that these mesophyll types disperse light throughout the tissue and could possibly lead to increased photosynthetic capacity [82], but future studies will hopefully elucidate the functional explanation for these mesophyll types in southern African *Oxalis*.

It is possible that the homogenous arrangement, with uniformly rounded mesophyll cells, could actually be immature and undifferentiated versions of isobilateral mesophyll due to heteroblastic development that is common among *Oxalis* species [83]. The morphology of heteroblasty in sect. *Ionoxalis* has been described, but to our knowledge no anatomical studies have assessed the internal differences between young and old *Oxalis* leaves [13]. For our study we strived to select only mature leaves, and it seems unlikely that such an argument could apply to two entire clades (Clades 11 and 13). For these reasons we favour the position that the isobilateral and homogenous mesophyll types are separate character states.

### Vascular tissue

*Oxalis* taxa with sheaths around their vascular bundles were distributed throughout the phylogeny, but all taxa from Clades 11 and 12 had these sheaths, which therefore form reliable diagnostic traits of these clades. Ancestral character state reconstruction analysis inferred high rates of change for this trait, resulting in equivocal reconstructions for most deep nodes. However, the most likely ancestral state to Clade 11 (and likely Clade 12: Table 1) was the presence of these sheaths.

Similar sheaths have been described around the periphery of the vascular bundles in the distantly related *O. corniculata* [51]. We can speculate that these sheaths have a functional significance, perhaps to give structural support to leaflets. However, if this was support tissue, expectedly this tissue should be lignified and we did not observe this in any of the studied leaflet material. Xylem and phloem arrangement and proportions in leaflet vascular tissue was more or less uniform in all studied taxa, and we could find no other phylogenetically significant data in transvers sections, although it is possible that leaflet venation patterns, which were only superficially explored in this study, could hold promise.

### Leaflet conduplication

Despite the relative subjectivity of assigning leaflets to the categories of flat vs. conduplicate, this trait showed some phylogenetic pattern, and defined the majority of southern African *Oxalis* clades. Conduplicate leaflets appear to be the most likely ancestral states to the majority of the well-supported clades, while flat leaflets were the reconstructed ancestral state to Clade 7b. To our knowledge this trait has not been considered to be taxonomically significant in other *Oxalis* species. Leaflet movements induced by light intensity, precipitation and wind speed [36] are most commonly linked to diurnal rhythms (where leaflets fold at night) in *Oxalis* [8, 13], but all studied taxa were sampled during the day. Ancestral character state reconstruction analysis showed that the leaflet type was equivocal to the remainder of the southern African *Oxalis* clades and that there were a few reversals between character states. The higher rates of change between these character states indicate that these characters evolve more quickly and consequently have less phylogenetic signal.

## Conclusions

A leaflet anatomical dataset of 110 southern African *Oxalis* species was compiled and used to assess variation of these traits in a phylogenetic context. The findings of this study showed that a combination of six leaflet anatomical traits (stomatal position, adaxial epidermal cell types, abaxial epidermal cell types, mesophyll type, presence or absence of a sheath around vascular tissue and degree of leaflet conduplication) clearly support various southern African *Oxalis* clades defined by previous DNA-based phylogenetic work [19]. The current character states among extant taxa and our knowledge of phylogenetic relationships among these taxa strongly reconstruct an ancestral southern African *Oxalis* that had irregular AD and semi-swollen AB epidermal cells on hypostomatic, bifacial leaflets. Major transitions in southern African *Oxalis* include the change from hypostomaty to epistomaty and the loss of semi-swollen AB epidermal cells in Clade 4, change from epistomatic to amphistomatic leaflets and the gain of swollen epidermal cells in Clade 7b, the change from bifacial mesophyll to isobilateral mesophyll in Clade 12 and homogenous mesophyll in Clades 11 and 13. Despite the phylogenetic patterns detected in the aforementioned traits, other leaflet anatomical traits were highly variable and showed no phylogenetic pattern (supplementary data). It is possible that these traits are evolving at elevated rates or are subject to substantial environmentally-induced variation, and may hold functional significance. The extensive leaflet anatomical dataset compiled through this study will aid in the taxonomic revision of this species-rich genus inhabiting of the Greater Cape Floristic Region and provide a basis for future hypotheses regarding its radiation.

## Additional files

Additional file 1: Table S1. Discrete and continuous leaflet anatomical data for southern African *Oxalis*. The discrete and continuous data of all studied leaflet anatomical traits. Captions of columns for discrete data include character state descriptions and captions of columns for continuous data include units (when applicable). (XLSX 143 kb)
Additional file 2: Table S2. The list of newly-collected taxa added to the phylogeny of Oberlander et al. (2011). Description of data: Phylogenetic trees were reconstructed using an expanded ITS data set from Oberlander et al. (2011) [19], including sequences from 13 newly-collected taxa. Species names, MO-accession numbers, source-origin and GenBank accession data are included. (XLSX 9 kb)
Additional file 3: Figure S1. A phylogenetic tree with additional discrete-data of leaflet anatomical traits observed in southern African *Oxalis*. A single ITS phylogenetic tree from the BEAST posterior distribution for southern African *Oxalis* taxa. The discrete data of 15 additional leaflet anatomical traits as noted in the results section of this article. (TIF 6750 kb)

## Abbreviations

AB: Abaxial
AD: Adaxial
ARD: All rates different
ER: Equal rates
GCFR: Greater Cape Floristic Region
ITS: Internal transcribed spacer
MCMC: Markov Chain Monte Carlo
ML: Maximum likelihood
SYM: Symmetrical
