# Supplementary figures and images for: The phylogenetic significance of leaf anatomical traits of southern African Oxalis
Source: BMC Evol Biol. 2016 Oct 22;16:225. doi: 10.1186/s12862-016-0792-z (PMC5075164; doi:10.1186/s12862-016-0792-z)

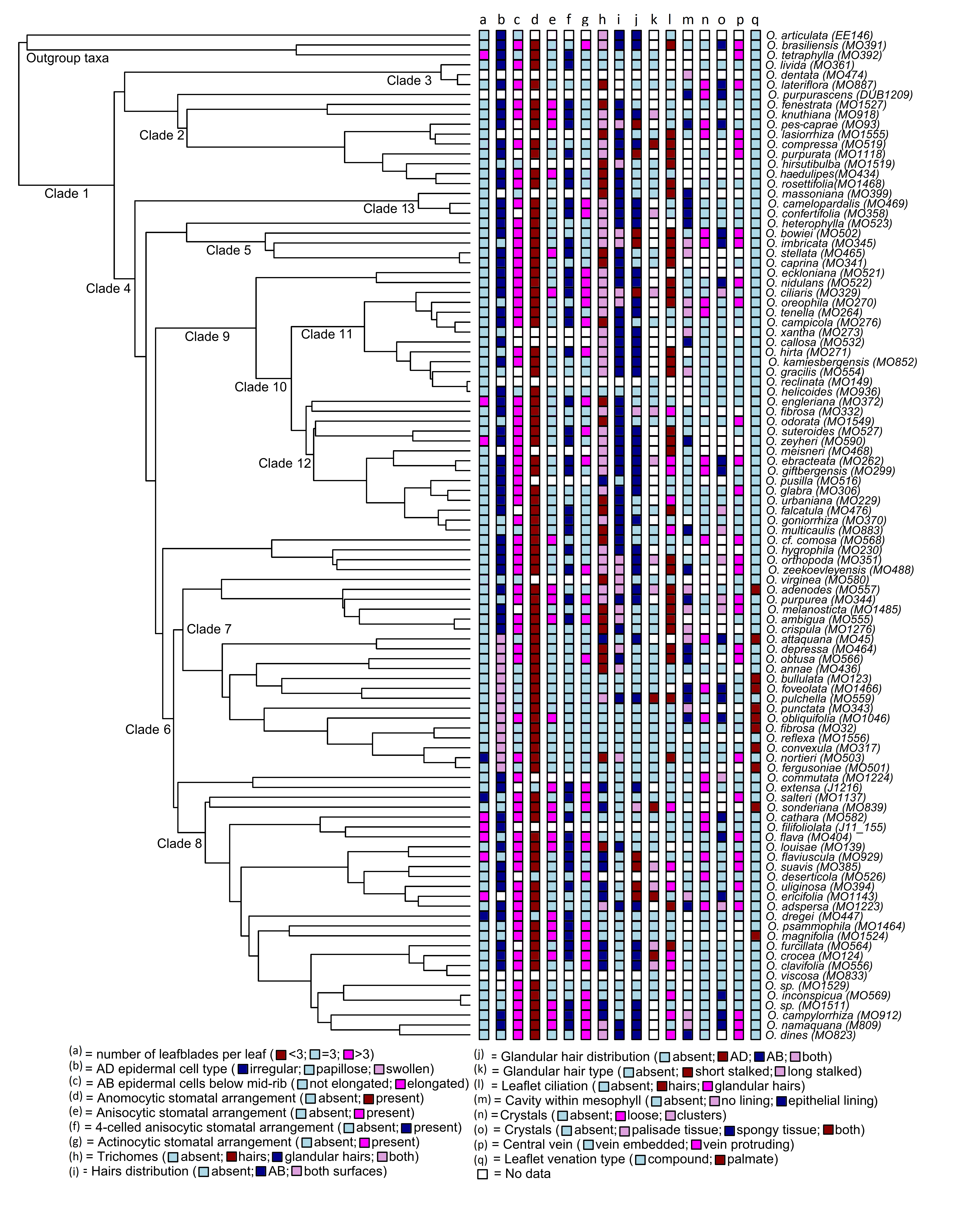

Supplement: Additional file 3: Figure S1. — A phylogenetic tree with additional discrete-data of leaflet anatomical traits observed in southern African Oxalis. A single ITS phylogenetic tree from the BEAST posterior distribution for southern African Oxalis taxa. The discrete data of 15 additional leaflet anatomical traits as noted in the results section of this article. (TIF 6750 kb) [file 12862_2016_792_MOESM3_ESM.tif]
